# In silico analysis and molecular identification of an anaphase-promoting complex homologue from human pathogen *Entamoeba histolytica*

**DOI:** 10.1186/s43141-021-00234-y

**Published:** 2021-09-01

**Authors:** Suchetana Pal, Pinaki Biswas, Raktim Ghosh, Somasri Dam

**Affiliations:** Department of Microbiology, The University of Burdwan , Burdwan, West Bengal 713104 India

**Keywords:** *Entamoeba histolytica*, APC, Homology modelling, In silico analysis, Molecular identification

## Abstract

**Background:**

Amoebiasis, being endemic worldwide, is the second leading cause of parasite-associated morbidity and mortality after malaria. The human parasite *Entamoeba histolytica* is responsible for the disease. Metronidazole is considered as the gold standard for the treatment of amoebiasis, but this antibiotic is carcinogenic and the development of antibiotic resistance against *E*. *histolytica* is a major health concern. Chromosome segregation is irregular in this parasite due to the absence of a few cell cycle checkpoint proteins. Anaphase-promoting complex (APC/C or cyclosome) is an E3 ubiquitin ligase that synchronizes chromosome segregation and anaphase progression via the ubiquitin-proteasome system. Proteasome is considered to be an attractive drug target for protozoan parasites. For the present study, EhApc11 from *E*. *histolytica*, a homologue of Apc11 in humans, is selected for elucidating its structural and functional aspects by detailed in silico analysis and molecular methods. Its physicochemical characteristics, identification of probable interactors, 3D model and quality analysis are done using standard bioinformatics tools. cDNA sequence of EhAPC11 has been further cloned for molecular characterization.

**Result:**

Conserved domain analysis revealed that EhApc11 belongs to the RING (really interesting new gene) superfamily and has ligand binding capacity. Expression study in *Escherichia coli* BL21 (DE3) revealed that the molecular weight of glutathione S-transferase (GST)-tagged protein is ~ 36 kDa.

**Conclusion:**

EhApc11 is a hydrophilic, thermostable, extracellular protein with potent antigenicity. The study will serve as a groundwork for future in-depth analysis regarding the validation of protein-protein interaction of EhApc11 with its substrates identified by STRING analysis and the potential of EhApc11 to serve as an anti-amoebic drug target.

**Supplementary Information:**

The online version contains supplementary material available at 10.1186/s43141-021-00234-y.

## Background

Amoebiasis, caused by the enteric protozoan parasite *Entamoeba histolytica*, is a major global health issue affecting about 50 million individuals around the world each year [1]. The disease is mostly endemic in developing countries with sub-optimal sanitization, improper water treatment and lower socio-economic status. Amoebiasis has been reported all over India, affecting about 15% of the Indian population. Microscopic examination is considered as the first choice of diagnosis till now [2]. Commercially available *E*. *histolytica* IgG antibody detection assay which is based on detecting anti-*E*. *histolytica* antibodies in the serum sample possesses a challenge in distinguishing past and current infections due to the persistence of IgG from past exposure to the pathogen [3]. Biochemical methods like isoenzyme analysis are not followed anymore due to poor sensitivity and a high rate of false-negative cases [4]. Commercially available TechLab *E*. *histolytica* II ELISA, a second-generation monoclonal antibody-based rapid test, revealed variable sensitivity and specificity patterns in several studies too [3, 5–7]. Real-time PCR has been reported to show higher diagnostic sensitivity than the antigen detection method [8]. However, high expenditure is still a barrier to the robustness of these molecular diagnostic methods in developing countries. Nonetheless, the tests developed so far for amoebiasis do not fit properly with the ASSURED criteria developed by the World Health Organization (WHO), which further intensifies the need for searching for a new drug target [9]. Metronidazole and other nitroimidazole derivatives remained the drug of choice in the treatment of amoebiasis for over 25 years. Yet, the persistence of the parasite in 40 to 60% of cases even after complete drug therapy has remained a lingering problem [10, 11]. Initially reported cases of drug resistance in the parasite were fairly uncommon, an attribute assumed to have resulted from the pleiotropic mode of action of metronidazole [12]. However, the eventual failure of differentiating pathogenic and non-pathogenic strains of *Entamoeba* has lead to indiscriminate use of these antibiotics and eventually drug resistance [13]. In vitro experiments have shown the survival capability of *E*. *histolytica* at 40 μM concentration of metronidazole which is ten times higher than its sublethal dosage [14]. The carcinogenic and teratogenic effects of metronidazole are debated over a long period. Later, carcinogenicity has been proved in rodents with high dosage treatment with extended duration, but in humans, the effect is still questioned [15]. However, a limited correlation between metronidazole intake and cancer was found in some cases, which eventually pushed the drug to officially be classified as ‘reasonably anticipated to be a human carcinogen’ [16]. Considering all these scenarios along with a lack of effective vaccine, the development of novel anti-amoebic compounds is the only choice to take on this pathogenic parasite [17].

Proteins have always been a forefront runner for being potential drug targets since their involvement in mediating several biological processes. Apart from that, efficient binding of selective compounds, being other desirable criteria, makes proteins a much better option than other macromolecules [18]. Cell cycles of protozoan parasites, having several unusual facets regarding the regulation of cell division, absence or altered checkpoint proteins, has gained a lot of attention as probable drug targets for quite a while [19, 20]. The existence of functional proteasome and components of the ubiquitin-proteasome system (UPS) has already been reported in *E*. *histolytica* [21, 22]. Ubiquitin-mediated proteolysis is an important process that governs several key regulatory cell cycle control events by degrading components of cell cycle machinery [20]. Polyubiquitination is a three-step cascade mechanism involving three groups of enzymes: ubiquitin-activating enzymes (E1), ubiquitin-conjugating enzymes (E2) and ubiquitin ligases (E3) [23]. Anaphase-promoting complex or cyclosome (APC/C) is a multi-subunit E3 ubiquitin ligase that spatiotemporally coordinates mitosis by controlling anaphase onset, progression to next steps, mitotic exit and re-entry into the next G1 phase by targeting specific substrates for 26S proteasome-mediated destruction, thereby controlling cell proliferation in all eukaryotes [24]. APC/C is made up of 12 subunits in humans and 13 subunits in budding yeast, but Apc2-Apc11 are considered to be a minimal ligase module essential for ubiquitination [25, 26]. There is no proper evidence of Apc11 acting as ubiquitin carrier onto substrates, but its role to act as a bridge between E2 and substrate has been reported to favour ubiquitination in *Saccharomyces cerevisiae* [27]. siRNA-induced apoptosis of cancer cells by targeting Cdc20 (an activator of APC/C) has been done effectively in humans [28]; therefore, it will be significant to understand if targeting the catalytic core of APC/C can be another option to arrest the cell cycle. Apc11 dysfunction and subsequent mitotic arrest have been reported previously [29]. The current study was undertaken to characterize EhApc11 that may shed light on its structural and functional aspects and can elucidate the prospect of EhApc11 to be considered as an anti-amoebic drug target and eventually provide insights toward the novel ones.

## Methods

### In silico analysis

#### Sequence retrieval

Human Apc11 protein sequence was retrieved from NCBI (NCBI Acc. No. NP_057560.8). The homologous protein of human Apc11 in *E. histolytica* was identified using human Apc11 as a query in BLASTP. The homologues from other representative model organisms were further identified using EhApc11 as a query sequence.

#### Multiple sequence alignment and phylogenetic analysis

Multiple sequence alignment of Apc11 from *E*. *histolytica* and other selected model organisms was performed by the ClustalW module of BioEdit v7.2.5. A phylogenetic tree was derived using the neighbour-joining algorithm of MEGA10 with 1000 bootstrap replicates.

#### Physicochemical profiling and primary structure analysis

Physicochemical characterization of the retrieved protein sequence was carried out using ExPASy’s ProtParam tool [30]. Several different parameters like molecular mass, theoretical isoelectric point (pI), amino acid composition, aliphatic index and grand average of hydropathy (GRAVY) were predicted which gives an idea about the intrinsic heterogeneity of the protein. The highest contributing amino acids (top five) in EhApc11 are compared. Under the default configuration, the analysis was done.

#### Secondary structure analysis

PSIPRED v4.0 was used to predict the secondary structure of EhApc11 in terms of the proportion of α-helices, strands and coils [31].

#### Tertiary structure analysis

Three-dimensional homology modelling of EhApc11 was performed by MODELLER [32]. Human APC/C-Cdc20-Cdk2-cyclinA2-Cks2 complex (PDB ID: 6Q6G chain C at 3.20 Å resolution) with a significant sequence identity was used as a template to model the tertiary structure of EhApc11. Validation of the modelled structure was performed by several bioinformatics servers viz., QMEAN, ProSA and ERRAT [33–35]. To find the energetically favourable residues within the modelled protein structure, PROCHECK was used to generate the Ramachandran plot which gives an overview of *φ*-*ψ* torsion angles of the protein backbone and also indicates the percentage of residues present within favoured, allowed and outlier regions [36].

#### Functional analysis and protein-protein interaction

STRING web server (https://string-db.org/) was used to identify in vivo interacting proteins of EhApc11. The prediction is based on both physical and functional associations [37]. However, the functional association between two proteins is considered as a basic unit in STRING where several sources including (1) known experimental and co-expression studies, (2) pathway knowledge from databases, (3) automated text mining, (4) information from computational algorithms and (5) pre-computed orthology relations are behind the derived prediction. The sequence was subjected to MEME for the identification of functional motifs or signature sequences [38]. For further analysis, NCBI-CDD-BLAST was used to find conserved domains present within the protein sequence.

#### Prediction of ligand binding site, subcellular localization and antigenicity

Protein-ligand binding sites were predicted using MODELLER-generated model as an input into COACH [39]. Final ligand binding sites are predicted by combining the results with COFACTOR, FINDSITE and ConCavity [39]. Subcellular localization of EhApc11 was predicted with CELLO v.2.5 as it is an important attribute to understand the protein functions and also carries a great significance in the drug designing process [40, 41]. Antigenic segments within EhApc11 were predicted with Predicted Antigenic Peptides (http://imed.med.ucm.es/Tools/antigenic.pl).

### Molecular characterization

#### Culture and maintenance of *E*. *histolytica*

The axenic cell culture of *E*. *histolytica* HM1:IMSS has been maintained. Trophozoites are grown in TYI-S-33 medium supplemented with 10% adult bovine serum at 37 °C, and log phase culture has been used for the present study [42].

#### RNA isolation and molecular cloning of full-length EhAPC11 cDNA

Total RNA was isolated from the trophozoites of *E*. *histolytica* HM1:IMSS using RNeasy Mini Kit (Qiagen) following the manufacturer’s protocol. cDNA was synthesized from 1 μg of total RNA sample using QuantiTect Reverse Transcription kit (Qiagen). The full-length cDNA fragment of EhAPC11 was PCR amplified using a specific set of primers (forward 5′AGC**GGATCC**ATGACTGTTGAAATAATTGAA-3′ and reverse 5′AGC**GTCGAC**ATTTTCTATTTCCATTAAATTT-3′). The following programme was used for amplification: 95 °C for 3 min, followed by 30 cycles of denaturation at 95 °C for 1 min, annealing at 50 °C for 45 s and final extension at 68 °C for 5 min. The PCR products were analysed in 1% agarose gel in 1× TAE buffer. The purified product was cloned into the *Bam*HI*-Sal*I sites of the bacterial expression vector, pGEX-4T-1, using *E*. *coli* XL1B competent cells and sequence verified by automated sequencing.

#### Expression and purification of recombinant EhApc11

The pGEX-4T-1:EhAPC11 was expressed in *E. coli* BL21 (DE3) as N-terminus GST-tagged fusion protein. Protein expression was induced with 0.1 mM isopropyl thio-β-galactoside (IPTG) for 16 h at 16 °C. Cells were harvested by centrifugation at 5000 rpm, and pellets were resuspended in lysis buffer containing 25 mM Tris-HCl pH 7.4, 10 mM NaCl, 5 mM MgCl_2_, 5 mM dithiothreitol, 1% Triton X-100 and 1 mM PMSF. Cell lysates were briefly sonicated to destroy the cell membrane and reduce the viscosity. Final centrifugation at 13,000 rpm for 20 min was followed by purification of supernatant fraction using GSH-agarose resin, and expression was analysed on 12% SDS-PAGE.

## Results

### Sequence retrieval and phylogenetic analysis

**Fig. 1:**
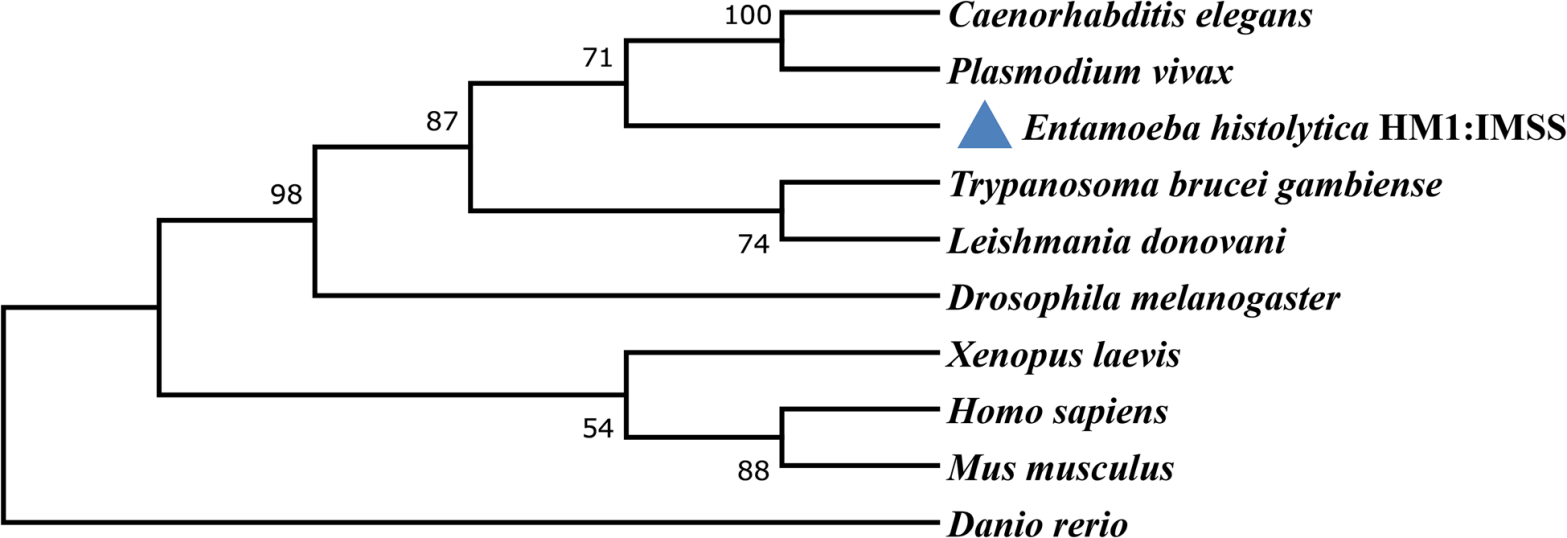
Phylogenetic analysis showing the evolutionary relationship of EhApc11 with other reference organisms using the neighbour-joining method of MEGA10. The numbers below and above the branch points are indicative of the confidence level of relationships as determined by bootstrap analysis

BLASTP analysis against the non-redundant database using *Homo sapiens* Apc11 (NP_057560.8) as query revealed that it shares significant sequence identity and similarity to zinc finger domain-containing protein (NCBI Acc. No. XP_651657.1) in *E. histolytica*. To better understand the conservation of Apc11 among other model organisms, the sequence of EhApc11 was used as a query in additional BLASTP searches. Details of EhApc11 protein and its homologous protein sequences considered for the phylogenetic study are provided in Table S1. The primary amino acid sequence of EhApc11 was aligned with homologues from other organisms, and strictly conserved residues were shaded in blue and identical residues were marked in black colour (Figure S1). Phylogenetic analysis revealed that EhApc11 is closer to other parasites, such as *Trypanosoma brucei gambiense*, *Plasmodium vivax* and *Leishmania donovani* whereas *Danio rerio* showed maximum divergence (Fig. 1).

### Primary structure analysis and physicochemical characterization

**Fig. 2:**
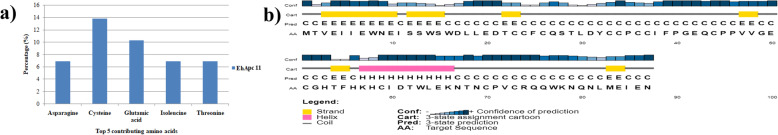
Primary and secondary structure analysis of EhApc11. **a** A column graph representing the amino acids dominating in the primary structure. **b** Secondary structural arrangement predicted by PSIPRED

Analysis of the physicochemical properties gives an overall idea about the nature of a protein. The number of amino acids, pI value (isoelectric point), aliphatic index and grand average of hydropathicity (GRAVY) of EhApc11 is given in Table 1. Computational analysis revealed that EhApc11 is 87 residues long, and its pI value is 4.40. The aliphatic index and GRAVY value of EhApc11 are found to be 62.64 and − 0.313, respectively. The primary structure analysis revealed cysteine as the most contributing amino acid (Fig. 2a). The percentage of the top five contributing amino acids of EhApc11 is represented in Fig. 2a.

**Table 1:** Physicochemical characterization of EhApc11

No. of amino acids	Molecular weight (kDa)	Theoretical pI	Aliphatic index	GRAVY
87	10.21	4.40	62.64	− 0.313

### Secondary structure analysis

The secondary structure analysis revealed that EhApc11 is rich in random coils, followed by strands and helices (Fig. 2b). Random coils have important functions in protein flexibility and allow conformational changes during enzymatic turnover [43].

### Tertiary structure analysis and model validation

**Fig. 3:**
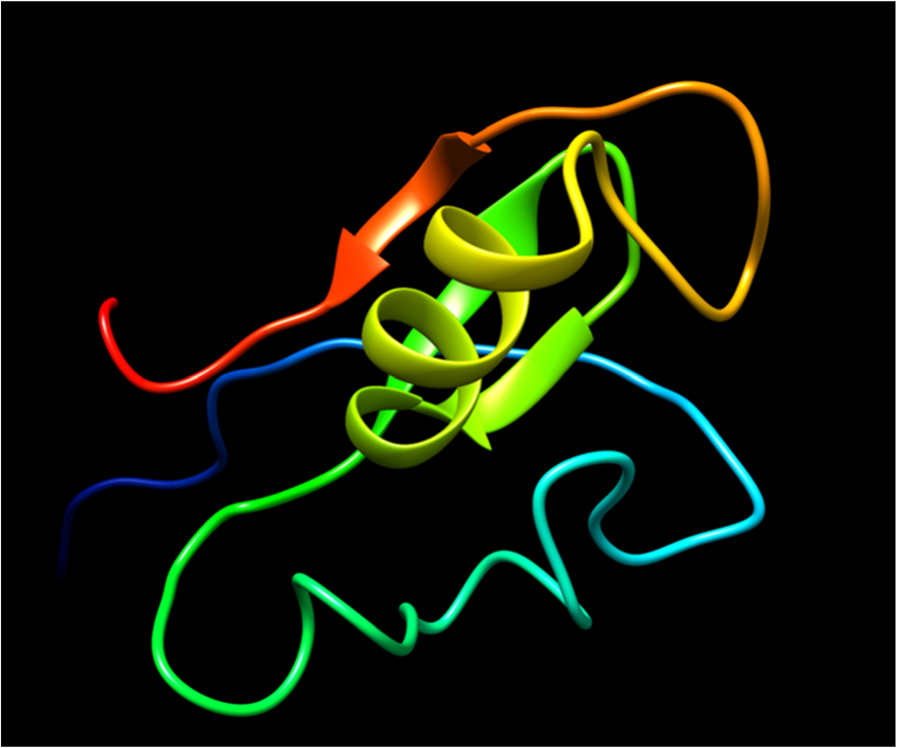
Modelled tertiary structure of EhApc11 protein. The structure prediction was done by MODELLER. Chimera was used for model visualization

**Fig. 4:**
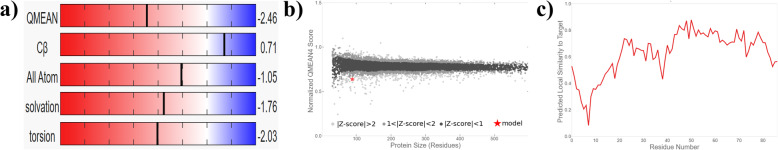
Quality assessment of modelled EhApc11 structure by QMEAN server. **a** Global quality estimation. **b** *z*-score value (− 2.46) of EhApc11 shown as a red star. **c** Local quality estimation. QMEAN scoring function depends on four individual components. Local geometry analysis is based on the potential of torsion angle over three consecutive amino acids. Long-range interactions are assessed at the residue level and at the second level based on the potential of Cβ atoms and all atoms, respectively. Solvation energy is calculated to know the accessibility of residues to water. The QMEAN *z*-score of − 4.0 or below is an indication of low model quality. The QMEAN *z*-score is shown on top, and individual *z*-scores are displayed below. The local quality plot is interpreted as the possibility of each residue of the model (on the *x*-axis) to be similar or deviable to the available native structures (on the *y*-axis). Residues with a score below 0.6 indicate deflection from expected values of native structures

Sequence similarity may correspond optimally to structural similarity, and knowledge of three-dimensional protein structures along with functional aspects may be helpful to elucidate the nature of uncharacterized proteins. The output model was visualized by Chimera [44] (Fig. 3). The stereochemical quality and accuracy of the modelled structure were validated through several tools (Table 2). The ERRAT analysis of non-bonded interactions between different atom types within EhApc11 indicated good model quality as the ERRAT score of EhApc11 was 84.375 (the accepted limit is > 50 for high-quality models) (Figure S2). Global quality estimation by QMEAN placed the EhApc11 model within the acceptable region of the estimated absolute model quality graph with a *z*-score of − 2.46 (Fig. 4a–c). The stereochemical evaluation of backbone *φ* and *ψ* dihedral angles of EhApc11 by PROCHECK revealed that 96.1% of residues were in the most favoured regions (Figure S3a). A good model is expected to have over 90% of residues in most favoured regions. ProSA was used to find potential errors present in the 3D model of the protein structure. It generates two characteristic features of input structures; *z*-score and a plot of its residue energies. The *z*-score signifies the overall model quality and assesses the structure’s total energy deviation from an energy distribution derived from random conformations [34, 45]. ProSA analysis revealed a *z*-score of − 3.21 for EhApc11 indicating no notable deflection from similar-sized typical native proteins (Figure S3b).

**Table 2:** Quality assessment scores of 3D modelled structure of EhApc11

QMEAN *z*-score	ProSA *z*-score	ERRAT quality factor (%)	Amino acids in favoured region of Ramachandran plot
− 2.46	− 3.21	84.375	96.1%

### Biological function prediction

**Fig. 5:**
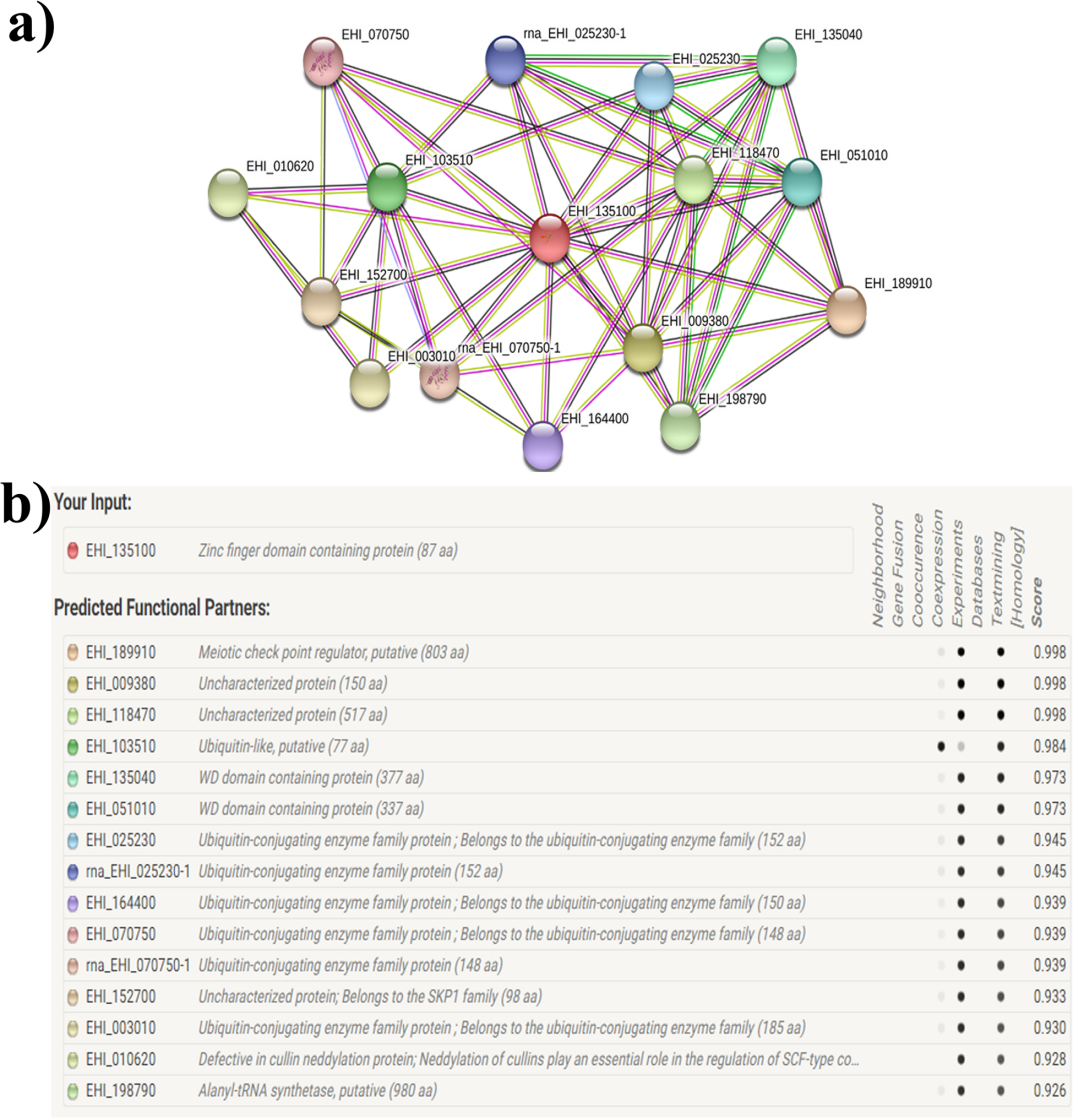
Protein-protein interactions of EhApc11 by STRING. **a** Network map of predicted interactions. **b** Summary of predicted interacting proteins. The network nodes indicate interacting proteins. The position of EhApc11 is labelled as EHI_135100. Seven different coloured lines indicate the seven different categories of evidences used in predicting the associations (green = neighbourhood, red = gene fusion, blue = co-occurrence, black = co-expression, pink = experiments, sky blue = databases, light green = text mining, grey = homology)

STRING analysis has revealed that EhApc11 is capable of interacting with a variety of proteins (Fig. 5). Alanyl-tRNA synthetase is an interactor of EhApc11 and has much relevance as aminoacyl-tRNA synthetases appear to be promising drug targets against eukaryotic parasites [46]. Another APC/C subunit [(EHI_009380), NCBI Acc. No. XP_654652.1], which is a homologue of human Apc10 (NP_055700.2), is predicted as a strong interactor of EhApc11. The MEME is a powerful web-based tool to identify and characterize sequence motifs in proteins. The location and position of the motifs within EhApc11 are depicted in Figure S4. The presence of a conserved domain can give us insights into the cellular or molecular function of a protein along with evolutionary history [47]. Conserved domains are considered as an important factor while assigning proteins into specific families. EhApc11 was found to be a member of the RING (really interesting new gene) superfamily with a ‘cross-brace’ motif. RINGs are cysteine and histidine-rich, zinc-binding domains reported to play a role in spatial coupling of E2 and E3 ubiquitin ligase while cross-brace arrangement helps in binding two atoms of zinc [48].

### Ligand binding site, localization and antigenicity prediction

**Fig. 6:**
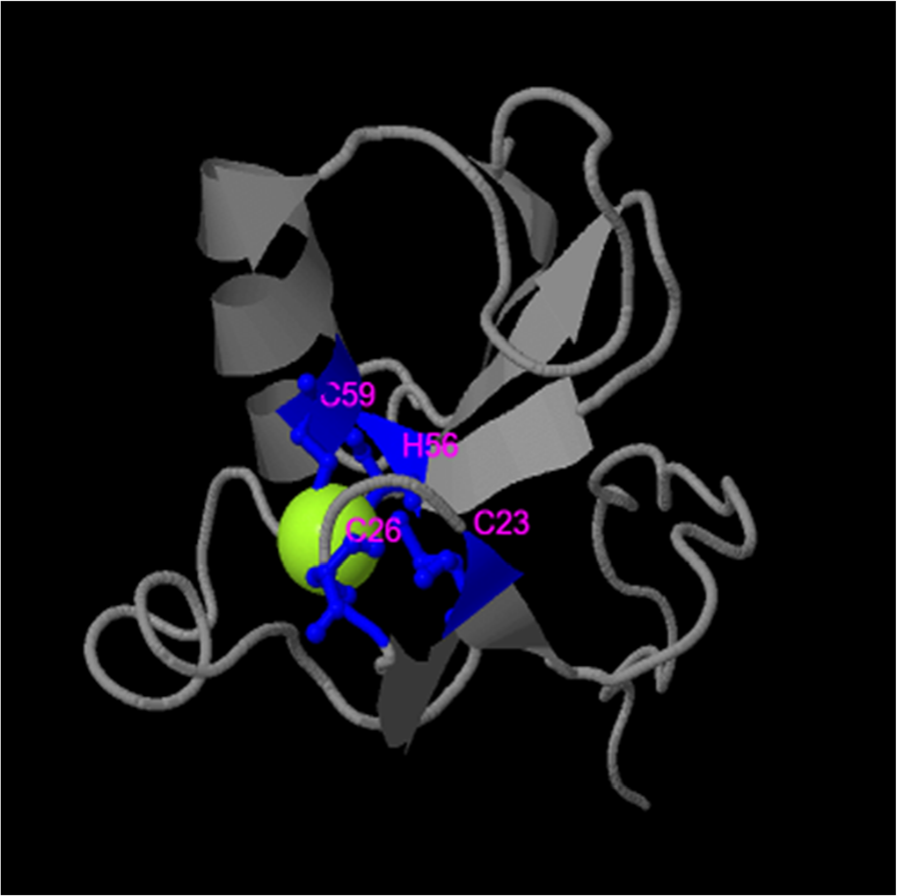
Ligand binding site prediction of EhApc11. COACH output indicates that zinc binds to a cysteine residue at positions 23, 26, and 59 and with a histidine residue at position 56 with a *C*-score of 0.75. Range values of *C*-score prediction lie between 0 and 1, where the highest score indicates more reliability

**Fig. 7:**
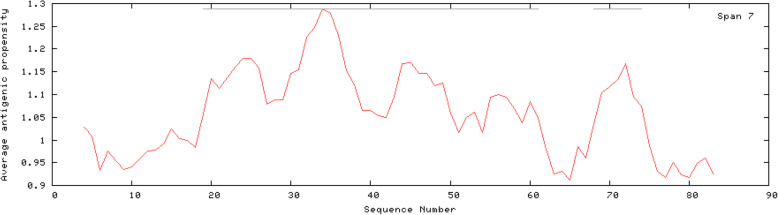
Antigenicity plot and antigenic determinants of EhApc11. Two antigenic determinants (spanning the regions 19–61 and 68–74) are indicated by grey lines within the plot

Proteins bind to other biomolecules or ions for participating in various essential biological processes. Specific key amino acid residues where interaction between proteins and ligands occur are termed as ligand binding site. Prediction of ligand binding sites is fundamental for the functional identification of a protein [49]. The amino acids around ligand binding sites are likely to be conserved among homologous proteins [50]. COACH analysis predicted a total of four ligand binding residues for zinc atoms at positions 23, 26, 56 and 59 with a significant support score (*C*-score) of 0.75 (Fig. 6). CELLO v.2.5 analysis predicted EhApc11 as an extracellular protein. EhApc11 was predicted as antigenic having an average antigenic propensity of 1.0513 (Table 3; Fig. 7).

**Table 3:** Antigenic determinants of EhApc11. The table indicates the positions and sequences that might be associated with the antigenic propensity of EhApc11

Number	Start position	Sequence(s)	End position
1	19	LEDTCCFCQSTLDYCCPCCIFPGEQCPPVVGECGHTFHKHCID	61
2	68	TNCPVCR	74

### Molecular characterization

**Fig. 8:**
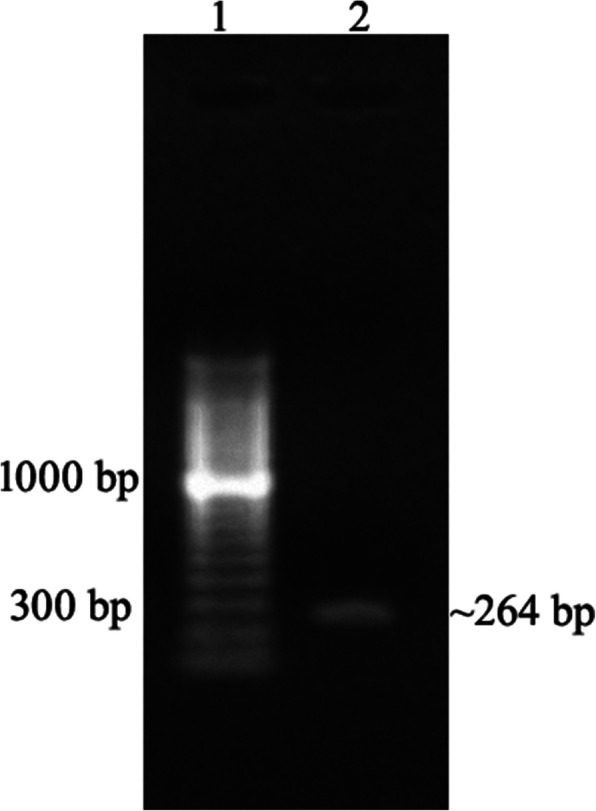
EhAPC11 cDNA amplicon separated by 1% agarose gel electrophoresis. Lane 1, 100 bp ladder ; Lane 2, PCR product of EhAPC11. The ~ 264-bp band represents EhAPC11 cDNA

**Fig. 9:**
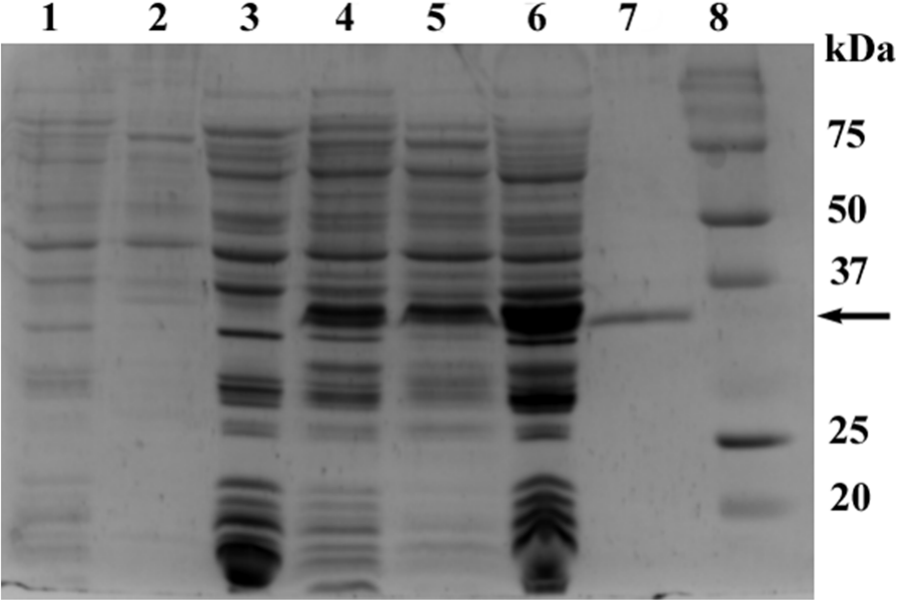
Expression and purification analysis of recombinant Apc11. Lane 1, uninduced 1 ml crude; lane 2, uninduced supernatant; lane 3, uninduced pellet; lane 4, induced 1 ml crude; lane 5, induced supernatant; lane 6, induced pellet; lane 7, purified protein; lane 8, molecular weight marker. A specific band around ~ 36 kDa indicates induced EhApc11 fusion protein

Amplification of EhAPC11 resulted in a PCR product of 264 bp (Fig. 8). A similar-sized specific product was obtained by *Bam*HI*-Sal*I double digestion of pGEX-4T-1: EhAPC11. The result concluded the proper cloning of EhAPC11 into pGEX-4T-1, supported by automated sequencing. With IPTG induction, an increase in protein expression is observed. The expressed protein identified by SDS-PAGE produced a specific band of ~ 36 kDa which corresponds to the predicted molecular weight of GST tagged recombinant protein (Fig. 9).

## Discussion

The aim of the present study was to gain structural and functional insights of EhApc11 using bioinformatic tools. The phylogenetic analysis highlights the conservation of the homologue proteins among reference organisms. The physicochemical properties of a protein are of much importance to predict systematic properties. EhApc11’s predicted pI (isoelectric point) value is less than 7, indicating that the protein is acidic. Aliphatic index is the relative volume of a protein occupied by aliphatic side chains. The high aliphatic index indicates the thermostability of a protein over a wide temperature range [51]. Therefore, an aliphatic index of 62.64 suggests the thermostable nature of EhApc11. The GRAVY value for a protein can be calculated as the sum of hydropathy values for all amino acids within a protein divided by the number of total residues in the sequence [52]. Negative GRAVY value is indicative of better interaction between protein and water. A GRAVY index of − 0.313 indicates the hydrophilic (globular) nature of EhApc11. Primary structure analysis revealed cysteine as the most contributing amino acid (Fig. 2a). Secondary structure analysis by PSIPRED showed that 65.51% of total amino acids contributed to coils, 11.49% to helices and 22.98% to strands. High percentage of random coil indicates true enzymatic functions of the protein. Theoretical structure prediction plays a key role in determining the function of an unknown protein. Structure prediction of EhApc11 was done successfully, and model quality assessment revealed that the predicted 3D model is of good stereochemical quality and accuracy. As one of the important properties of RING superfamily protein, EhApc11 is rich in cysteine and histidine which is supported by multiple sequence alignment data. Nearly 200 RING proteins have been discovered to be associated with oncogenesis, apoptosis, transcriptional regulation and ubiquitination [48]. RING finger proteins are the largest class of E3s discovered so far [53]. Members of the RING family are thought to aid in the formation of large molecular assemblies. RING domain is present in all RING E3s and activates E2-ubiquitin conjugates, thus allowing ubiquitin to be transferred directly from E2 to the target protein. Mutation of *Saccharomyces cerevisiae* Roc1 (ScRoc1), a homologue of Apc11 protein, has been reported to abolish the ligase activity of the complex [54]. It indicates the functional conservation of Apc11 to maintain E3 ubiquitin ligase activity. STRING analysis revealed that EhApc11 can potentially interact with various proteins involved in the cell cycle pathway and also with EhApc10. This is significant because it has been reported that human Apc10 may regulate the interaction between Apc2-Apc11 [55]. Proteins having confidence scores above 0.9 were included in the result (maximum 20 interactors in 1st shell is considered). The extracellular nature of EhApc11 is a positive aspect as extracellular proteins are always considered as good drug targets due to their accessibility. The presence of ligand binding sites for zinc atoms reveals the conserved nature of the RING domain present in EhApc11. The ligation of zinc is essential for proper folding of the domain and subsequent biological functions. The identification of protein antigenic epitopes aids in the identification of protein areas capable of generating a robust immune response that may be significant for producing antibodies. Our bioinformatic analysis revealed two regions that might be associated with antigenicity of EhApc11. However, further research is needed to determine the applicability in this aspect. The SDS-PAGE analysis showed that the recombinant EhApc11 was properly expressed in bacteria.

## Conclusion

UPS plays a key role in maintaining cellular homeostasis. Being a part of UPS, APC/C holds much importance as an E3 ubiquitin ligase in regulating cell cycle events through controlled proteolysis of specific cell cycle proteins and may also serve as a potential anti-amoebic target. Thus, analysis of the structural and functional properties of EhApc11 will provide more clarity. In silico analysis revealed that EhApc11 is a hydrophilic, extracellular protein with a molecular weight of ~ 10.21 kDa. The predicted 3D structure may help in the further in-depth analysis of protein-protein interactions and docking studies, as crystal structure-related details of EhApc11 were scarce. The extracellular nature of EhApc11 makes it easily accessible to the host immune system. The protein possesses two antigenic determinants and belongs to the RING superfamily. The expression study in *E. coli* BL21 (DE3) shows the molecular weight of GST tagged EhApc11 is ~ 36 kDa. While the study has provided basic insights into the structural and functional aspects of the protein, detailed experiments regarding the validation of protein-protein interaction as identified by STRING and phenotypic analysis of Apc proteins would help to get new knowledge about this human pathogen. This ultimately would help to identify new therapeutic targets against amoebiasis in the near future.

## Supplementary Information

**Additional file 1: Table S1.** Details of Apc11 homologs from different organisms along with their query coverage, percentage identity and accession number.

**Additional file 2: Figure S1.** Multiple sequence alignment showing degree of conservation of Apc11 amino acid sequences from different organisms. Identical residues are shaded in blue whereas similar residues are presented in black colour. Cysteine is observed as the most conserved amino acid followed by histidine, tryptophan, phenyl alanine and isoleucine.

**Additional file 3: Figure S2.** ERRAT verification plot for the modelled EhApc11 structure. Regions that can be rejected as 95% confidence level are in yellow colour. The overall quality factor of the modelled protein is 84.375.

**Additional file 4: Figure S3.** Validation of EhApc11 model through PROCHECK and ProSA. a) Ramachandran plot obtained through PROCHECK revealed 96.1% of the total amino acid residues within most favoured regions. b) ProSA z-score value of EhApc11 was -3.21, located within the characteristic range of native proteins from different sources (NMR and X-ray).

**Additional file 5: Figure S4.** Conserved motif analysis in EhApc11.MEME was used to predict conserved motifs and maximum number parameter was set to 3.

## Data Availability

Not applicable.

## Abbreviations

UPS: Ubiquitin-proteasome system
APC: Anaphase-promoting complex
RING: Really interesting new gene

## References

[1] Bercu TE, Petri WA, Behm JW (2007). Amebic colitis: new insights into pathogenesis and treatment. Curr Gastroenterol Rep.

[2] Fotedar R, Stark D, Beebe N, Marriott D, Ellis J, Harkness J (2007). Laboratory diagnostic techniques for *Entamoeba* species. Clin Microbiol Rev.

[3] Zeehaida M, Wan Nor Amilah WAW, Amry AR, Hassan S, Sarimah A, Rahmah N (2008). A study on the usefulness of Techlab *Entamoeba histolytica* II antigen detection ELISA in the diagnosis of amoebic liver abscess (ALA) at Hospital Universiti Sains Malaysia (HUSM), Kelantan, Malaysia. Trop Biomed.

[4] Haque R, Ali IK, Akther S, Petri WA (1998). Comparison of PCR, isoenzyme analysis, and antigen detection for diagnosis of *Entamoeba histolytica* infection. J Clin Microbiol.

[5] Gatti S, Swierczynski G, Robinson F, Anselmi M, Corrales J, Moreira J (2002). Amebic infections due to the *Entamoeba histolytica*-*Entamoeba dispar* complex: a study of the incidence in a remote rural area of Ecuador. Am J Trop Med Hyg.

[6] Visser LG, Verweij JJ, Van Esbroeck M, Edeling WM, Clerinx J, Polderman AM (2006). Diagnostic methods for differentiation of *Entamoeba histolytica* and *Entamoeba dispar* in carriers: performance and clinical implications in a non-endemic setting. Int J Med Microbiol IJMM.

[7] Mohanty S, Sharma N, Deb M (2014). Microscopy versus enzyme linked immunosorbent assay test for detection of *Entamoeba histolytica* infection in stool samples. Trop Parasitol.

[8] Ahmad N, Khan M, Hoque MI, Haque R, Mondol D (2007). Detection of *Entamoeba histolytica* DNA from liver abscess aspirate using polymerase chain reaction (PCR): a diagnostic tool for amoebic liver abscess. Bangladesh Med Res Counc Bull.

[9] Mabey D, Peeling RW, Ustianowski A, Perkins MD (2004). Diagnostics for the developing world. Nat Rev Microbiol.

[10] Irusen EM, Jackson TF, Simjee AE (1992). Asymptomatic intestinal colonization by pathogenic *Entamoeba histolytica* in amebic liver abscess: prevalence, response to therapy, and pathogenic potential. Clin Infect Dis Off Publ Infect Dis Soc Am.

[11] Spillmann R, Ayala SC, Sanchez CE (1976). Double-blind test of metronidazole and tinidazole in the treatment of asymptomatic *Entamoeba histolytica* and *Entamoeba hartmanni* carriers. Am J Trop Med Hyg.

[12] Leitsch D (2019). A review on metronidazole: an old warhorse in antimicrobial chemotherapy. Parasitology.

[13] Bansal D, Malla N, Mahajan RC (2006). Drug resistance in amoebiasis. Indian J Med Res.

[14] Wassmann C, Hellberg A, Tannich E, Bruchhaus I (1999). Metronidazole resistance in the protozoan parasite *Entamoeba histolytica* is associated with increased expression of iron-containing superoxide dismutase and peroxiredoxin and decreased expression of ferredoxin 1 and flavin reductase. J Biol Chem.

[15] Dobiás L, Cerná M, Rössner P, Srám R (1994). Genotoxicity and carcinogenicity of metronidazole. Mutat Res.

[16] Friedman GD, Jiang S-F, Udaltsova N, Quesenberry CP, Chan J, Habel LA (2009). Epidemiologic evaluation of pharmaceuticals with limited evidence of carcinogenicity. Int J Cancer.

[17] Stanley SL (2006). Vaccines for amoebiasis: barriers and opportunities. Parasitology.

[18] Xu H, Xu H, Lin M, Wang W, Li Z, Huang J, Chen YZ, Chen X (2007). Learning the drug target-likeness of a protein. Proteomics.

[19] Banerjee S, Das S, Lohia A (2002). Eukaryotic checkpoints are absent in the cell division cycle of *Entamoeba histolytica*. J Biosci.

[20] Grant KM (2008). Targeting the cell cycle in the pursuit of novel chemotherapies against parasitic protozoa. Curr Pharm Des.

[21] Sánchez R, Alagón A, Stock RP (2002). *Entamoeba histolytica*: intracellular distribution of the proteasome. Exp Parasitol.

[22] Arya S, Sharma G, Gupta P, Tiwari S (2012). In silico analysis of ubiquitin/ubiquitin-like modifiers and their conjugating enzymes in *Entamoeba* species. Parasitol Res.

[23] Ciechanover A, Iwai K (2004). The ubiquitin system: from basic mechanisms to the patient bed. IUBMB Life.

[24] Sudakin V, Ganoth D, Dahan A, Heller H, Hershko J, Luca FC, Ruderman JV, Hershko A (1995). The cyclosome, a large complex containing cyclin-selective ubiquitin ligase activity, targets cyclins for destruction at the end of mitosis. Mol Biol Cell.

[25] Peters J-M (2006). The anaphase promoting complex/cyclosome: a machine designed to destroy. Nat Rev Mol Cell Biol.

[26] Tang Z, Li B, Bharadwaj R, Zhu H, Ozkan E, Hakala K (2001). APC2 Cullin protein and APC11 RING protein comprise the minimal ubiquitin ligase module of the anaphase-promoting complex. Mol Biol Cell.

[27] Leverson JD, Joazeiro CA, Page AM, Huang HK, Hieter P, Hunter T (2000). The APC11 RING-H2 finger mediates E2-dependent ubiquitination. Mol Biol Cell.

[28] Huang H-C, Shi J, Orth JD, Mitchison TJ (2009). Evidence that mitotic exit is a better cancer therapeutic target than spindle assembly. Cancer Cell.

[29] Chang T-S, Jeong W, Lee D-Y, Cho C-S, Rhee SG (2004). The RING-H2-finger protein APC11 as a target of hydrogen peroxide. Free Radic Biol Med.

[30] Wilkins MR, Gasteiger E, Bairoch A, Sanchez JC, Williams KL, Appel RD (1999). Protein identification and analysis tools in the ExPASy server. Methods Mol Biol Clifton NJ.

[31] Buchan DWA, Jones DT (2019). The PSIPRED Protein Analysis Workbench: 20 years on. Nucleic Acids Res.

[32] Webb B, Sali A (2016). Comparative protein structure modeling using MODELLER. Curr Protoc Bioinforma.

[33] Benkert P, Künzli M, Schwede T (2009). QMEAN server for protein model quality estimation. Nucleic Acids Res.

[34] Wiederstein M, Sippl MJ (2007). ProSA-web: interactive web service for the recognition of errors in three-dimensional structures of proteins. Nucleic Acids Res.

[35] Colovos C, Yeates TO (1993). Verification of protein structures: patterns of nonbonded atomic interactions. Protein Sci Publ Protein Soc.

[36] Laskowski RA, MacArthur MW, Moss DS, Thornton JM (1993). PROCHECK: a program to check the stereochemical quality of protein structures. J Appl Crystallogr.

[37] Szklarczyk D, Franceschini A, Wyder S, Forslund K, Heller D, Huerta-Cepas J, Simonovic M, Roth A, Santos A, Tsafou KP, Kuhn M, Bork P, Jensen LJ, von Mering C (2015). STRING v10: protein-protein interaction networks, integrated over the tree of life. Nucleic Acids Res.

[38] Bailey TL, Boden M, Buske FA, Frith M, Grant CE, Clementi L, Ren J, Li WW, Noble WS (2009). MEME Suite: tools for motif discovery and searching. Nucleic Acids Res.

[39] Yang J, Roy A, Zhang Y (2013). Protein-ligand binding site recognition using complementary binding-specific substructure comparison and sequence profile alignment. Bioinforma Oxf Engl.

[40] Yu C-S, Chen Y-C, Lu C-H, Hwang J-K (2006). Prediction of protein subcellular localization. Proteins.

[41] Rajendran L, Knölker H-J, Simons K (2010). Subcellular targeting strategies for drug design and delivery. Nat Rev Drug Discov.

[42] Diamond LS, Harlow DR, Cunnick CC (1978). A new medium for the axenic cultivation of *Entamoeba histolytica* and other *Entamoeba*. Trans R Soc Trop Med Hyg.

[43] Buxbaum E (2015) Fundamentals of protein structure and function, 2nd edn. Springer International Publishing 10.1007/978-3-319-19920-7

[44] Pettersen EF, Goddard TD, Huang CC, Couch GS, Greenblatt DM, Meng EC, Ferrin TE (2004). UCSF Chimera--a visualization system for exploratory research and analysis. J Comput Chem.

[45] Sippl MJ (1993). Recognition of errors in three-dimensional structures of proteins. Proteins.

[46] Pham JS, Dawson KL, Jackson KE, Lim EE, Pasaje CFA, Turner KEC, Ralph SA (2014). Aminoacyl-tRNA synthetases as drug targets in eukaryotic parasites. Int J Parasitol Drugs Drug Resist.

[47] Marchler-Bauer A, Lu S, Anderson JB, Chitsaz F, Derbyshire MK, DeWeese-Scott C, Fong JH, Geer LY, Geer RC, Gonzales NR, Gwadz M, Hurwitz DI, Jackson JD, Ke Z, Lanczycki CJ, Lu F, Marchler GH, Mullokandov M, Omelchenko MV, Robertson CL, Song JS, Thanki N, Yamashita RA, Zhang D, Zhang N, Zheng C, Bryant SH (2011). CDD: a Conserved Domain Database for the functional annotation of proteins. Nucleic Acids Res.

[48] Borden KL (2000). RING domains: master builders of molecular scaffolds?. J Mol Biol.

[49] Kinoshita K, Nakamura H (2005). Identification of the ligand binding sites on the molecular surface of proteins. Protein Sci Publ Protein Soc.

[50] Magliery TJ, Regan L (2005). Sequence variation in ligand binding sites in proteins. BMC Bioinformatics.

[51] Ikai A (1980). Thermostability and aliphatic index of globular proteins. J Biochem (Tokyo).

[52] Kyte J, Doolittle RF (1982). A simple method for displaying the hydropathic character of a protein. J Mol Biol.

[53] Chasapis CT, Spyroulias GA (2009). RING finger E(3) ubiquitin ligases: structure and drug discovery. Curr Pharm Des.

[54] Ohta T, Michel JJ, Schottelius AJ, Xiong Y (1999). ROC1, a homolog of APC11, represents a family of cullin partners with an associated ubiquitin ligase activity. Mol Cell.

[55] Buschhorn BA, Petzold G, Galova M, Dube P, Kraft C, Herzog F, Stark H, Peters JM (2011). Substrate binding on the APC/C occurs between the co-activator CDH1 and the processivity factor DOC1. Nat Struct Mol Biol.

