# Supplementary material for: In silico analysis and molecular identification of an anaphase-promoting complex homologue from human pathogen Entamoeba histolytica
Source: J Genet Eng Biotechnol. 2021 Sep 1;19:133. doi: 10.1186/s43141-021-00234-y (PMC8410921; doi:10.1186/s43141-021-00234-y)
Supplement: Supplementary file 1 — Additional file 1: Table S1. Details of Apc11 homologs from different organisms along with their query coverage, percentage identity and accession number. [file 43141_2021_234_MOESM1_ESM.doc]

**Table S1.** Details of Apc11 homologs from different organisms along with their query coverage, percentage identity and accession number

| **Sl no.** | **Organism(s)** | **Query coverage** | **Percentage identity** | **Accession number** |
| --- | --- | --- | --- | --- |
| 1 | *Trypanosoma brucei gambiense* | 89% | 46.91% | XP_011776779.1 |
| 2 | *Leishmania donovani* | 89% | 39.51% | XP_003865034.1 |
| 3 | *Plasmodium vivax* | 88% | 29.67% | XP_001614522.1 |
| 4 | *Caenorhabditis elegans* | 88% | 40.96% | NP_497937.1 |
| 5 | *Xenopus laevis* | 89% | 44.44% | NP_001165194.1 |
| 6 | *Drosophila melanogaster* | 89% | 41.98% | NP_001097122.1 |
| 7 | *Danio rerio* | **89%** | **44.44%** | NP_001091950.1 |
| 8 | *Mus musculus* | **89%** | **43.21%** | NP_001033319.1 |
