# Supplementary figures and images for: In silico analysis and molecular identification of an anaphase-promoting complex homologue from human pathogen Entamoeba histolytica
Source: J Genet Eng Biotechnol. 2021 Sep 1;19:133. doi: 10.1186/s43141-021-00234-y (PMC8410921; doi:10.1186/s43141-021-00234-y)

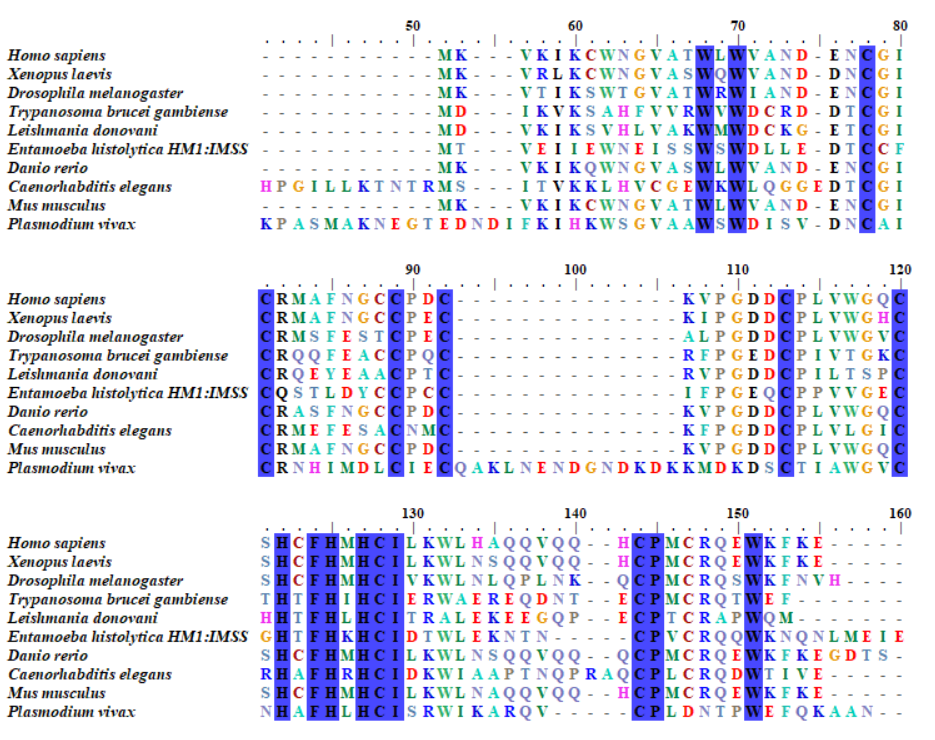

Supplement: Supplementary file 2 — Additional file 2: Figure S1. Multiple sequence alignment showing degree of conservation of Apc11 amino acid sequences from different organisms. Identical residues are shaded in blue whereas similar residues are presented in black colour. Cysteine is observed as the most conserved amino acid followed by histidine, tryptophan, phenyl alanine and isoleucine. [file 43141_2021_234_MOESM2_ESM.tif]

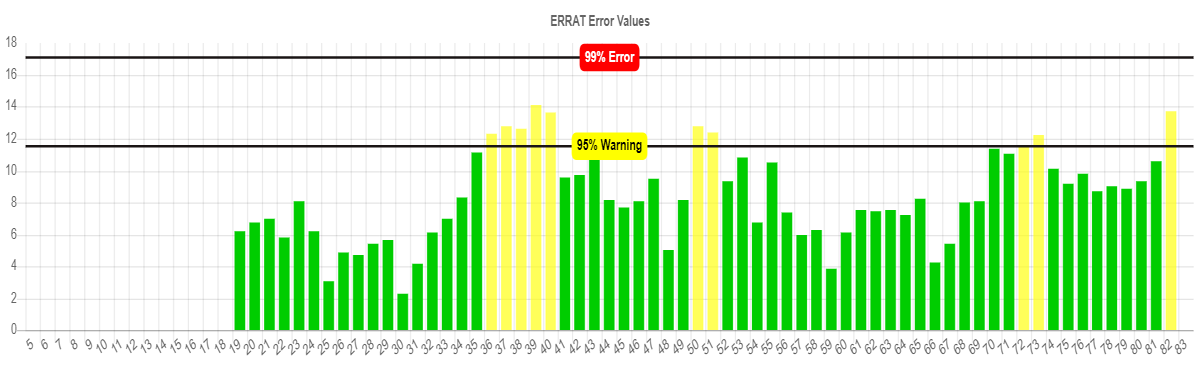

Supplement: Supplementary file 3 — Additional file 3: Figure S2. ERRAT verification plot for the modelled EhApc11 structure. Regions that can be rejected as 95% confidence level are in yellow colour. The overall quality factor of the modelled protein is 84.375. [file 43141_2021_234_MOESM3_ESM.tif]

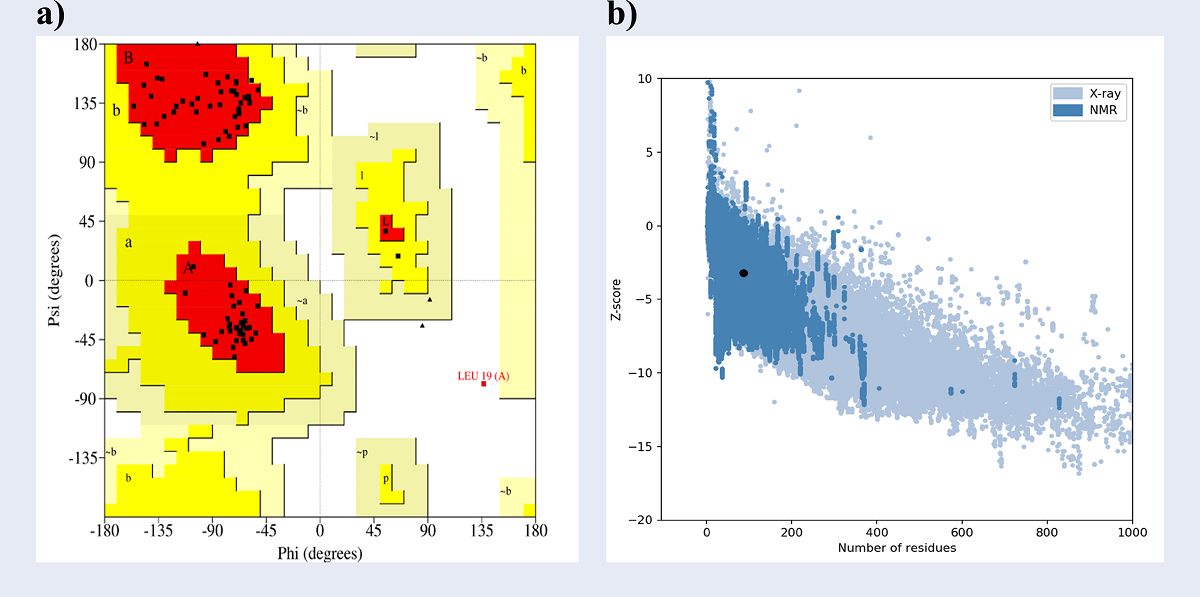

Supplement: Supplementary file 4 — Additional file 4: Figure S3. Validation of EhApc11 model through PROCHECK and ProSA. a) Ramachandran plot obtained through PROCHECK revealed 96.1% of the total amino acid residues within most favoured regions. b) ProSA z-score value of EhApc11 was -3.21, located within the characteristic range of native proteins from different sources (NMR and X-ray). [file 43141_2021_234_MOESM4_ESM.tif]

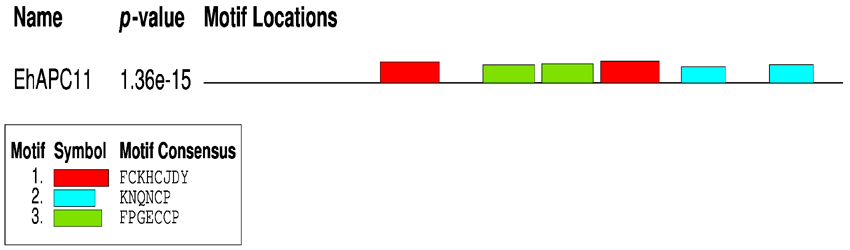

Supplement: Supplementary file 5 — Additional file 5: Figure S4. Conserved motif analysis in EhApc11.MEME was used to predict conserved motifs and maximum number parameter was set to 3. [file 43141_2021_234_MOESM5_ESM.tif]
